# Efficacy and safety of Yingxin pill for stable angina pectoris with heart blood stasis obstruction syndrome: a randomized, single-blind, positive-controlled trial

**DOI:** 10.3389/fphar.2026.1730842

**Published:** 2026-02-17

**Authors:** Chaofeng Niu, Haixia Lai, Ce Wang, Zhuoran Wu, Peiyu Zhang, Lan Wei, Wang Sun, Juwei Dong, Liyong Ma, Yujie Shi, Lijing Zhang

**Affiliations:** 1 Department of Cardiology, Dongzhimen Hospital, Beijing University of Chinese Medicine, Beijing, China; 2 Department of Integrated Traditional Chinese and Western Medicine, Hohhot Maternal and Child Health Hospital, Hohhot, China; 3 Department of Cardiology, The Seventh Medical Center of Chinese PLA General Hospital, Beijing, China

**Keywords:** blood lipid, inflammatory markers, positive-controlled clinical trial, stable angina pectoris, traditional Chinese medicine, Yingxin pill

## Abstract

**Objective:**

To evaluate the clinical efficacy and safety of Yingxin Pill (YXP) in patients with stable angina pectoris (SAP) and heart blood stasis obstruction syndrome.

**Methods:**

Sixty patients were randomly assigned to either the experimental group (YXP plus conventional Western medicine) or the control group (Shexiang Baoxin Pill [SBP] plus conventional Western medicine), with 30 patients in each group. Treatment lasted for 4 weeks. Outcomes included the Seattle Angina Questionnaire (SAQ), a Traditional Chinese Medicine (TCM) efficacy scale, blood lipid profiles, and inflammatory markers. The effective rate and incidence of adverse events were also compared.

**Results:**

After treatment, both groups showed significant improvements in SAQ scores, TCM efficacy scale scores, lipid profiles, and inflammatory markers compared to baseline (P < 0.05). There were no significant differences between the two groups in these outcomes, nor in the total effective rate or incidence of adverse events (P > 0.05).

**Conclusion:**

The addition of YXP to conventional therapy can improve symptoms, reduce blood lipid and inflammation levels, and is safe for patients with SAP and heart blood stasis obstruction syndrome.

**Clinical Trial Registration:**

https://itmctr.ccebtcm.org.cn/, identifier ITMCTR2025000274.

## 1 Introduction

According to the Global Burden of Disease Report 2021, from 1990 to 2021, ischemic heart disease has always ranked first in the global mortality rate, and the global disability-adjusted life years increased from 159.9 million to 188.3 million (GBD, 2021 Causes of Death Collaborators et al., 2024; GBD, 2021 Diseases and Injuries Collaborators, 2024). The American Heart Association’s Heart and Stroke Statistics Report 2024 shows that, based on the data from the National Health and Nutrition Examination Survey (NHANES) from 2017 to 2020, the overall prevalence of cardiovascular disease (CVD) among adults aged 20 and above in the United States is 7.1%, and the overall prevalence of angina pectoris is 3.9% (Martin et al., 2024). The China HEART study adopted the ABM (Agent-Based Model) model to predict that by 2030, the incidence rate of CVD will rise from 0.74% in 2021 to 0.97% (Wang et al., 2025), and the mortality rate is expected to increase from 0.39% in 2021 to 0.46% in 2024. CVD remain the primary burden on China’s medical and health care. Stable angina pectoris (SAP) is a symptom of chest pain caused by insufficient blood supply in the coronary arteries based on fixed stenosis of the coronary arteries. It is often manifested as a crushing pain or a feeling of suffocation, with a short duration, and can be induced by exercise, emotional fluctuations, or other stress factors, and it can be relieved by rest or taking nitrates (Joshi and de Lemos, 2021). The treatment principles of SAP in Western medicine mainly focus on improving prognosis and relieving symptoms. In clinical practice, lipid-lowering drugs and antiplatelet drugs are commonly used to prevent adverse cardiovascular events, and beta-blockers, nitrates, or calcium channel blockers are used to improve the symptoms of angina pectoris.

In the field of Traditional Chinese medicine (TCM), SAP belongs to the category of “chest paralysis and heartache”, and its pathogenesis is summarized as “yang and micro yin strings” in the “Synopsis of the Golden Chamber”, that is, the yang in the chest is sluggish, and the cold yin invades internally, resulting in heart and pulse paralysis, it reveals that this disease belongs to the pattern of deficiency in origin and excessiveness in manifestation (O’Brien and Vitetta, 2012; Zhang et al., 2014). Regarding the distribution of syndromes, some scholars have found through data mining that SAP commonly presents with syndromes such as qi deficiency and blood stasis, intermingling of phlegm and blood stasis, and qi stagnation and blood stasis. The main treatment methods are promoting blood circulation to remove blood stasis, regulating qi to relieve stagnation, resolving phlegm and lowering turbidity, and replenishing qi to dredge the meridians (Ying et al., 2021; Liu and Huang, 2016).

With the development of medicine, TCM is widely used to treat angina pectoris, effectively improving the clinical symptoms and prognosis of patients. As early as 2020, CVD was listed as one of the dominant diseases in the treatment of proprietary Chinese medicines in the Guidelines for the Clinical Application of Proprietary Chinese Medicines in the Treatment of Dominant Diseases. Yingxin pill (YXP) has the effect of nourishing the heart and pulse, removing blood stasis, and relieving pain, it can improve myocardial ischemia and alleviate the symptoms of angina. To further clarify the clinical value of YXP, this study adopted a non-inferiority design and selected Shexiang Baoxin Pill (SBP), a widely recognized agent for SAP that has been validated by large-scale clinical trials, as the positive control (Wei et al., 2022; Wang et al., 2021; Ge et al., 2021). It aimed to systematically evaluate the efficacy of YXP combined with conventional Western medicine in the treatment of stable angina pectoris and to explore its effects on inflammatory indicators and blood lipid levels. By demonstrating the non-inferiority of YXP, the findings are intended to support its potential as an alternative treatment option, thereby expanding therapeutic choices for clinicians and patients.

## 2 Materials and methods

### 2.1 Study drug

#### 2.1.1 Botanical material and authentication

The YXP used in this study is a commercially available, multi-botanical drug TCM preparation. Its formulation consists of seven medicinal materials. The taxonomic identification of all botanical and zoological source materials was verified using the Kew Medicinal Plant Names Services (MPNS, http://mpns.kew.org/mpns-portal/) to ensure nomenclatural accuracy. The authenticated materials are as follows: Panax ginseng C. A. Mey. [Araliaceae; Ginseng Radix et Rhizoma], Syzygium aromaticum (L.) Merr. & L.M.Perry [Myrtaceae; Caryophylli Flos], Neolitsea cassia (L.) Kosterm. [Lauraceae; Cinnamomi Cortex], Bovis Calculus Artifactus [Artificial Bovis Calculus], Bufonis Venenum [Bufonidae; secretio glandularum auricularium et cutaneum *Bufo gargarizans*], Borneolum Syntheticum [Synthetic Borneolum], and Suis Fellis Pulvis [Suidae; Fellis Pulvis *Sus scrofa* domestica]. All materials were procured as a single batch (Lot No.: 20081492) by Beijing Tongrentang Science and Technology Development Co., Ltd., in compliance with the specifications of the Pharmacopoeia of the People’s Republic of China (2020 Edition) or the associated Drug Standards of the Ministry of Public Health. (see Supplementary Material).

#### 2.1.2 Formulation, preparation, and critical parameters

The manufacturing of YXP strictly adheres to the national drug standard published in the Drug Standards of Ministry of Public Health: Traditional Chinese Patent Medicine Formulations, Volume 15 (Standard No. WS3-B-2989-98). As a regulated, market-authorized product, the exact proportional ratios of its metabolite botanical drugs are fixed and supervised by the National Medical Products Administration. While the public standard focuses on quality control methods and does not disclose exact quantities per metabolite, a common practice for protecting proprietary manufacturing processes, it mandates a critical quantitative parameter: the ethanol-soluble extractives must be no less than 15.0% (w/w). This requirement provides a mandatory, macro-level quantitative control of the total chemical output of the fixed formulation, ensuring fundamental batch-to-batch consistency in the chemical profile.

Preparation Process: The production follows the standard protocol: specific botanical drugs are mixed with starch and pulverized; Bufonis Venenum is pulverized separately; Borneolum Syntheticum is finely ground. All powders are then triturated with vinegar, sieved, homogenized, and formed into pills using water as the binding agent. The pills are dried at a low temperature and subsequently coated and polished.

Drug-Extract Ratio (DER) and Dosage: As a water-pilled preparation where powdered crude drugs are directly formed into pills, the DER is approximately 1:1. The clinical dosage is 1-2 pills per administration (Specification: 1.5 g per 100 pills), twice daily. This study utilized a single, consistent product batch (Lot No.: 20081492), guaranteeing that all participants received a chemically uniform intervention.

#### 2.1.3 Chemical profiling and quality control system

YXP is governed by a rigorous and standardized quality control system that complies with the national pharmacopoeial standards and the ConPhyMP guidelines. Batch-to-batch chemical consistency is ensured through multiple complementary approaches, including thin-layer chromatography identification, determination of extractable matter, and quantitative assays of specific marker constituents. The complete details and validation of this quality control system have been comprehensively documented in previously published literature (Li, 1994).

Thin-Layer Chromatography (TLC) Fingerprint Identification: The standard specifies two specific TLC identifications: (a) for Bufonis Venenum using resibufogenin as a reference standard, and (b) for Suis Fellis Pulvis using cholic acid and hyodeoxycholic acid as reference standards. This serves as Orthogonal Method 1, providing qualitative confirmation of specific chemical metabolite groups.

Extractive Determination: The standard stipulates that ethanol-soluble extractives ≥15.0%. This serves as Orthogonal Method 2, providing macro-quantitative control of the overall chemical composition.

Quantitative Assay of Raw Material: An annex to the standard details a quantitative assay for the raw material Suis Fellis Pulvis, requiring a cholic acid content ≥45.0%. This serves as Orthogonal Method 3, providing precise quantitative control of a key metabolite.

Manufacturer’s Internal Control: The manufacturer employs modern analytical techniques such as High-Performance Liquid Chromatography (HPLC) within its Good Manufacturing Practice (GMP) framework for batch-release monitoring of metabolites like ginsenosides, thereby further ensuring chemical consistency.

#### 2.1.4 A priori safety assessment

YXP is an approved drug (Approval Number: Z11020151) with a history of clinical use in China spanning several decades, which informs its safety profile. Throughout the trial, all participants concurrently received standardized conventional Western medicine. Any adverse events were closely monitored and systematically recorded, with no drug-related serious adverse events reported in this study.

### 2.2 Study design

A total of 60 patients diagnosed with stable angina pectoris with the syndrome of heart blood stasis in the outpatient or inpatient department of Dongzhimen Hospital, Beijing University of Chinese Medicine from April 2023 to April 2024 were included. They were randomly divided into the experimental group and the control group at a ratio of 1:1, and the intervention continued for 4 weeks. All subjects participating in the study had signed the informed consent form before enrollment. The whole process of this study strictly followed the standards of the Declaration of Helsinki and the Good Clinical Practice (GCP). This study has been approved by the Medical Ethics Committee of Dongzhimen Hospital, Beijing University of Chinese Medicine (Ethics Approval Number: 2023DZMEC-010). It has been registered on the International Traditional Medicine Clinical Trial Registry Platform (Registration Number: ITMCTR2025000274).

### 2.3 Western medicine diagnostic criteria

The Western medical diagnostic criteria for CVD were formulated (Liu et al., 2024): (1) Coronary angiography of coronary arteries CT showed that the diameter of the lumen of the main coronary artery or its main branches was narrowed by ≥ 50%; (2) Percutaneous coronary intervention (PCI) or coronary artery bypass grafting (CABG). The diagnostic criteria for SAP (Joshi and de Lemos, 2021): (1) There was no significant change in the degree, frequency, nature, and predisposing factors of colic attacks in the past 6 0 days; (2) TnT/I negative, combined with electrocardiogram to exclude acute coronary syndrome.

### 2.4 TCM syndrome diagnostic criteria

The diagnostic criteria for heart blood stasis obstruction syndrome were referred to the Guidelines for the Diagnosis and Treatment of Stable Angina Pectoris with cardiovascular disease published by the Cardiovascular Disease Branch of the Chinese Association of Traditional Chinese Medicine in 2019, the scores are added to ≥8 Diagnosis can be made by points: (1) fixed chest pain (4 points); (2) Purple and dark tongue or ecchymosis and petechiae on the tongue (4 points); (3) sublingual vein purple dark (3 points); (4) Purple and dark complexion (3 points); (5) Petechiae or ecchymosis on the body (3 points); (6) Limb numbness (2 points); (7) Purple or dark red lips (2 points); (8) Pulse astringency (2 points).

### 2.5 Inclusion criteria

The inclusion criteria were as follows: (1) Meeting the diagnostic criteria for stable angina pectoris of CVD with the syndrome of heart blood stasis; (2) Aged between 18 and 85 years old, regardless of gender; (3) With the Canadian Cardiovascular Society (CCS) classification of angina pectoris at grade II - III; (4) Not having taken other traditional Chinese medicines or Chinese patent medicines within 2 weeks; (5) Subjects voluntarily participating in this study, being fully informed and signing the informed consent form.

### 2.6 Exclusion criteria

Patients who met one or more the following criteria were excluded: (1) The presence of high bleeding risk; (2) The presence of severe organic heart diseases, such as left ventricular ejection fraction (LVEF) <35% or New York Heart Association (NYHA)/Killip cardiac function classification at grade IV; (3) Those with a history of congenital heart disease or malignant tumors and other conditions that the researchers deemed unable to participate in the trial; (4) Severe liver or kidney insufficiency (alanine aminotransferase (ALT)or aspartate aminotransferase (AST) ≥3 × ULN, or creatinine clearance (Ccr) <30 mL/min); (5) Complicated with severe infections; (6) Females who were pregnant or in the lactation period; (7) Those with a history of alcohol abuse within the past 6 months; (8) Those with a history of drug abuse or drug dependence within 1 year before screening; (9) Those allergic to the metabolites of the experimental drugs; (10) Patients with mental disorders; (11) Other circumstances that the researchers considered inappropriate for participating in this trial.

### 2.7 Randomization and blinding

This study employed a random number table for randomization, with patients allocated in a 1:1 ratio to either the YXP group or the SBP group. Given the inherent differences in color, shape, and odor between the two investigational drugs (YXP and SBP), blinding of investigators and outcome assessors was not feasible. Therefore, a single-blind design (only participants were blinded) was adopted. To ensure the validity of participant blinding, both drugs were repackaged into uniform, opaque, and visually identical neutral packaging, with only random codes marked on the outside. The entire randomization list was maintained by independent personnel not involved in participant interaction. The dispensing and recording of medications were strictly carried out by a designated drug dispenser in accordance with this list, thereby ensuring allocation concealment and the implementation of participant blinding.

### 2.8 Sample size estimation

This study is a preliminary, exploratory non-inferiority randomized controlled trial, with the primary aim of generating initial evidence and hypotheses. Based on the practical feasibility of a single-center study and in reference to preliminary investigations of similar scale, a total of 60 patients (30 per group) were planned for analysis. With reference to literature data comparing SBP with placebo (Ge et al., 2021), the common standard deviation (σ) of the primary outcome measure (SAQ Angina Frequency score) was estimated. We considered this sample size adequate for a preliminary assessment of the efficacy and safety trends of YXP combined with conventional Western medicine in treating SAP, as well as for providing estimates of effect size and variability for future large-scale confirmatory studies.

### 2.9 Interventions

Based on the conventional Western medicine treatment, the experimental group was given YXP, while the control group was given SBP. Both groups continued to take the medications for 4 weeks.

YXP was manufactured by Beijing Tongrentang Science and Technology Development Co., Ltd., with the approval number of National Drug Approval Z11020151. SBP was manufactured by Shanghai Hutchison Pharmaceuticals Co., Ltd., with the approval number of National Drug Approval Z31020068. Its medicinal metabolites Moschus, Bovis Calculus Artifactus, extract of Ginseng Radix et Rhizoma, Styrax, Cinnamomi Cortex, Bufonis Venenum, and Borneolum Syntheticum.

### 2.10 Primary outcomes

In this study, the Seattle Angina Questionnaire (SAQ) was taken as the primary outcome. The SAQ illustrates the patient’s quality of life in five dimensions (physical limitation, anginal stability, anginal frequency, treatment satisfaction, and disease perception/quality of life) (Ge et al., 2021). All SAQ domain scores and the summary score range from 0 to 100 with higher scores indicating less angina, fewer functional limitations, and better quality of life (Spertus et al., 1995; Thomas et al., 2021). Observe the changes in the SAQ before treatment and 4 weeks after medication.

### 2.11 Secondary outcome

The secondary outcomes included TCM efficacy evaluation, total cholesterol (TC), Triglycerides (TG), low-density lipoprotein cholesterol (LDL-C), high-density lipoprotein cholesterol (HDL-C), small dense lipoprotein (HDL-C), low-density lipoprotein (sdLDL-C), lipoprotein a [lipoprotein(a), Lp(a)], high sensitivity C-reactive protein (hs-CRP), and interleukin-6 (IL-6).

The TCM efficacy evaluation was referred to the Guidelines for Clinical Research of New Drugs in Traditional Chinese Medicine, the clinical efficacy evaluation was divided into four levels according to the scoring results of the TCM Efficacy Evaluation Scale (Xing et al., 2021): (1) Significant efficacy: the score after treatment was higher than that before treatment 70% reduction ≥; (2) Effective: the score after treatment is reduced by ≥30% but <70% compared with before treatment; (3) Ineffective: the score after treatment is reduced by <30% compared with before treatment; (4) Aggravation: The score after treatment is reduced by <0 compared with before treatment.

### 2.12 Safety evaluation

Safety outcomes were evaluated in terms of vital signs, blood routine, biochemistry, blood coagulation, urine routine, stool routine, fecal occult blood test, electrocardiogram, and cardiac ultrasound. During the trial, outpatient or telephone follow-up visits were conducted every 2 weeks to record adverse reactions, incidence of adverse events and severity of adverse events.

### 2.13 Statistical analysis

Use SPSS 25.0 and R 4.4.0 statistical software to analyze the clinical data adopted in this study. Normally distributed continuous data are presented as mean ± standard deviation (Mean ± SD) and the summary statistics for pre-to post-treatment changes are reported as mean ± standard error (Mean ± SE), which is also used in relevant figures where appropriate. Comparisons between groups were performed using the independent samples t-test, while comparisons within groups before and after treatment were performed using the paired t-test. Non-normally distributed continuous data are presented as median (interquartile range) [M (IQR)], with comparisons between groups performed using the Mann-Whitney U test and comparisons within groups (pre-vs. post-treatment) performed using the Wilcoxon signed-rank test. Categorical data are presented as number (percentage) [n (%)] and were compared between groups using the chi-square test or Fisher’s exact test, as appropriate. All tests were two-sided, and a P-value <0.05 was considered statistically significant.

## 3 Result

### 3.1 Baseline characteristics of participants

**FIGURE 1:**
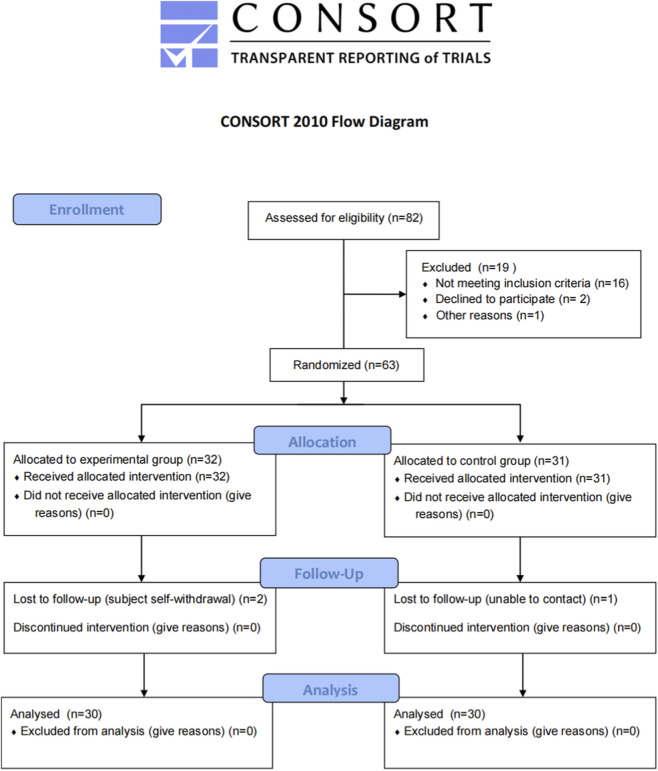
Participant flow diagram (CONSORT 2010).

A total of 82 participants were screened for this study, of whom 19 were excluded (16 did not meet the inclusion criteria, 2 declined to participate, and 1 for other reasons). The remaining 63 participants were randomized to the experimental group (n = 32) or the control group (n = 31), and all received the assigned intervention. During follow-up, two participants in the experimental group withdrew voluntarily and one participant in the control group was lost to follow-up. Ultimately, 30 participants in the experimental group and 30 in the control group completed the study and were included in the final analysis, resulting in an overall follow-up completion rate of 95.2%. The dataset was complete and suitable for efficacy and safety assessment (see Figure 1).

Baseline characteristics, including demographics, clinical features reflecting disease severity, and comorbidities, were compared between the two groups. Statistical analysis revealed no significant differences between the groups (*P* > 0.05), as illustrated in Table 1. This confirms the effectiveness of randomization and ensures the reliability of subsequent efficacy comparisons.

**TABLE 1:** Baseline characteristics data of participants.

Variables	YXP (n = 30)	SBP (n = 30)	*P* value
Demographics			
Age (years, Mean ± SD)	60.03 ± 12.94	64.53 ± 10.82	0.190
Gender (male) [n (%)]	25 (83.33%)	19 (63.33%)	0.080
Height (cm, Mean ± SD)	171.43 ± 7.98	168.30 ± 8.61	0.108
Weight (kg, Mean ± SD)	71.99 ± 9.79	68.83 ± 11.31	0.200
Clinical characteristics			
Disease course (years, Mean ± SD)	5.37 ± 4.99	7.20 ± 8.35	0.495
Angina frequency [episodes/week, M (IQR)]	5.0 (3.0, 8.0)	5.5 (3.3, 9.0)	0.561
Angina duration [minutes/episode, M (IQR)]	8.5 (6.0, 12.3)	9.0 (6.5, 13.0)	0.587
Nitroglycerin use [tablets/week, M (IQR)]	3.0 (2.0, 5.0)	3.0 (1.0, 5.3)	0.954
Concomitant disease [n (%)]			
Hypertension	20 (43.50%)	26 (56.50%)	0.067
Diabetes	16 (51.60%)	15 (48.40%)	0.796
Arrhythmia	10 (55.60%)	8 (44.40%)	0.573
Cerebrovascular disease	7 (63.60%)	4 (36.40%)	0.317

Data are presented as n (%), Mean ± SD, or M (IQR); YXP, yingxin pill; SBP, shexiang baoxin pill; Continuous variables, Independent t-test (normal) or Mann-Whitney U test (non-normal). Categorical variables, Chi-square or Fisher’s exact test. A P value <0.05 was considered statistically significant.

### 3.2 Primary outcomes

After 4 weeks of treatment, through data analysis and comparison, it was found that all dimensions of the SAQ in both groups had significantly improved compared with those before treatment (*P* < 0.05). Between-group comparisons revealed that the SBP group showed significantly greater improvement in angina frequency compared to the YXP group. Conversely, the YXP group demonstrated significantly greater improvement in anginal stability compared to the SBP group. No statistically significant between-group differences were observed in the dimensions of physical limitation, treatment satisfaction, or disease perception (*P* > 0.05). See Table 2 for details.

**TABLE 2:** Changes in SAQ Scores from Baseline After Treatment (Mean ± SE, n = 30).

SAQ Domain	YXP (n = 30)	SBP (n = 30)	Between-Group Difference (95% CI)	*P*-value (Between Groups)
Physical limitation	6.95 ± 0.85^*^	9.01 ± 0.90^*^	−2.06 (−4.51 to 0.39)	0.097
Anginal stability	41.67 ± 1.89^‡^	34.49 ± 2.01^‡^	7.18 (1.99–12.37)	0.007
Anginal frequency	9.33 ± 1.12^*^	15.86 ± 1.05^‡^	−6.53 (−9.39 to −3.67)	0.001
Treatment satisfaction	10.42 ± 1.05^*^	9.91 ± 0.98^*^	0.51 (−2.15–3.17)	0.703
Disease perception	13.34 ± 1.24^†^	10.34 ± 1.18^†^	3.00 (−0.39–6.39)	0.082

SAQ, seattle angina questionnaire; YXP, yingxin pill; SBP, shexiang baoxin pill; CI, confidence interval. Data are Mean ± SE (change from baseline). Between-group P-values compare score changes (t-test or Mann-Whitney U test). Within-group significance vs. baseline: *P < 0.05, †P < 0.01, ‡P < 0.001 (paired t-test or Wilcoxon test). Positive between-group difference (YXP–SBP) favors YXP.

### 3.3 Secondary outcome

#### 3.3.1 TCM efficacy

The TCM efficacy evaluation scale used in this study referred to the “Evaluation Standards for TCM Efficacy in Angina Pectoris of cardiovascular disease” issued by the Cardiovascular Disease Branch of the China Association of Traditional Chinese Medicine in 2018. The lower the score, the better the TCM efficacy. Data analysis showed that before and after treatment, there was no statistically significant difference in the scores of the TCM efficacy evaluation scale between the two groups (*P* > 0.05); the scale scores of each group after treatment had significantly changed compared with those before treatment (*P* < 0.001). See Table 3 for details.

**TABLE 3:** Comparison of Changes in TCM Syndrome Scores Before and After Treatment Between Two Groups [M (IQR), n = 30].

Group	Pre-treatment Score	Post-treatment Score	Change in Score	Between-group Difference (95% CI)	*P*-value (Between Groups)
YXP (n = 30)	36.00 (28.00,41.00)	25.00 (19.25,29.00)^‡^	−11.00 (−15.75, −6.00)	−0.50 (−6.21, 5.21)	0.861
SBP (n = 30)	39.00 (24.50,45.00)	23.50 (13.75,35.25)^‡^	−15.50 (−25.25, −8.75)		

YXP, yingxin pill; SBP, shexiang baoxin pill; M, median; IQR, interquartile range; CI, confidence interval. Data are M (IQR) for score changes (negative = improvement). Within-group: ‡P < 0.001 vs. baseline (Wilcoxon test). Between-group: Mann-Whitney U test; positive difference favors YXP.

In terms of the effectiveness evaluation, although the total effective rate of the YXP group was lower than that of the SBP group, there was no statistically significant difference between the groups (*P* > 0.05). See Table 4 for details.

**TABLE 4:** Comparison of TCM efficacy between the two groups [n (%)].

Group	Significant Effective	Effective	Ineffective	Aggravation	Total Effective Rate	*P*-value
YXP (n = 30)	2 (6.66%)	21 (70.00%)	7 (23.33%)	0 (0.00%)	23 (76.66%)	0.220
SBP (n = 30)	3 (10.00%)	23 (76.66%)	4 (13.33%)	0 (0.00%)	26 (86.66%)	

YXP, yingxin pill group; SBP, Shexiang Baoxin Pill group. Total Effective Rate = (Number of Markedly Effective + Number of Effective)/Total number × 100%. Between-group comparison was performed using the Fisher’s exact test.

#### 3.3.2 Clinical symptoms efficacy

After 4 weeks of treatment, both groups showed significant improvements from baseline in angina clinical symptoms and nitroglycerin use. Between-group comparisons indicated no statistically significant differences in the degree of improvement for any of the clinical symptom indicators (*P* > 0.05). Specifically, the between-group difference in the reduction of angina frequency was 0.50 episodes/week (95% CI: 0.20 to 1.20, P = 0.156), the between-group difference in the shortening of angina duration was 0.50 min/episode (95% CI: 0.52 to 1.52, P = 0.329), and the between-group difference in the reduction of nitroglycerin use was 0.20 tablets/week (95% CI: 0.42 to 0.82, P = 0.519). See Table 5 for details.

**TABLE 5:** Comparison of Changes in Clinical Symptoms After Treatment Between the Two Groups (Mean ± SE, n = 30).

Index	YXP (n = 30)	SBP (n = 30)	Between-Group Difference (95% CI)	*P*-value (Between Groups)
Change in angina frequency (episodes/week)	−3.00 ± 0.25^*^	−3.50 ± 0.30^‡^	0.50 (−0.20–1.20)	0.156
Change in angina duration (minutes/episode)	−4.20 ± 0.35^‡^	−4.70 ± 0.38^‡^	0.50 (−0.52–1.52)	0.329
Change in nitroglycerin use (tablets/week)	−2.00 ± 0.20^‡^	−2.20 ± 0.25^‡^	0.20 (−0.42–0.82)	0.519

YXP, yingxin pill; SBP, shexiang baoxin pill; CI, confidence interval. Data: mean change from baseline ±SE (negative = improvement). Within-group significance: *P < 0.05; †P < 0.01; ‡P < 0.001. Between-group: t-test orMann-Whitney U test, reporting mean difference (95% CI).

#### 3.3.3 Blood lipid

Data analysis showed that after treatment, the levels of TC, TG, HDL-C, LDL-C, sdLDL-C, and Lp(a) in both groups were better than those before treatment. There was no statistically significant difference between groups (*P* > 0.05). Among them, the level of Lp(a) in each group had significantly improved after treatment compared with that before treatment (*P* < 0.05). See Table 6 for details.

**TABLE 6:** Comparison of Blood Lipid Between Two Groups [M (IQR), n = 30].

Lipid Parameter	Group	Pre-treatment [M (IQR)]	Post-treatment [M (IQR)]	*P*-value
TC (mmol/L)	YXP	3.575 (2.955, 4.088)	3.200 (2.737, 3.685)	0.058
	SBP	3.305 (2.942, 3.595)	3.180 (2.575, 3.635)	0.065
TG (mmol/L)	YXP	1.240 (0.840, 1.627)	1.170 (0.920, 1.472)	0.465
	SBP	1.195 (0.975, 1.935)	1.190 (0.787, 1.570)	0.320
HDL-C (mmol/L)	YXP	1.060 (0.915, 1.185)	1.110 (0.927, 1.307)	0.130
	SBP	1.075 (0.952, 1.300)	1.050 (0.967, 1.302)	0.780
LDL-C (mmol/L)	YXP	1.930 (1.542, 2.438)	1.790 (1.515, 2.107)	0.062
	SBP	1.740 (1.570, 2.080)	1.665 (1.413, 2.030)	0.085
sdLDL-C (nmol/L)	YXP	0.620 (0.467, 0.862)	0.525 (0.435, 0.610)	0.053
	SBP	0.570 (0.422, 0.710)	0.525 (0.362, 0.707)	0.210
LP(a) (mg/L)	YXP	182.350 (136.970, 271.570)	125.200 (103.000, 199.800)	0.001^*^
	SBP	192.800 (125.550, 340.480)	120.250 (84.550, 222.530)	0.001^*^

YXP, yingxin pill; SBP, shexiang baoxin pill; Data, median (IQR). Abbreviations defined. Within-group pre-post comparison by Wilcoxon signed-rank test. Significance: *P < 0.05.

#### 3.3.4 Inflammatory marker

Data analysis showed that the levels of hs-CRP and IL-6 in both groups after treatment had significantly improved compared with those before treatment (*P* < 0.05). There was no statistically significant difference in the comparison between the groups (*P* > 0.05). See Table 7 for details.

**TABLE 7:** Comparison of hs-CRP and IL-6 between two groups [M (IQR), n = 30].

Lipid Parameter	Group	Pre-treatment [M (IQR)]	Post-treatment [M (IQR)]	*P*-value
hs-CRP (mg/L)	YXP	1.070 (0.790, 1.920)	0.850 (0.670, 1.365)	0.025^*^
	SBP	1.120 (0.920, 2.580)	0.915 (0.532, 1.890)	0.028^*^
TG (mmol/L)	YXP	2.640 (1.170, 5.190)	1.550 (0.887, 2.897)	0.008^*^
	SBP	2.486 (1.235, 6.222)	1.580 (1.082, 3.607)	0.012^*^

YXP, yingxin pill; SBP, shexiang baoxin pill; Data, M (IQR). Within-group pre-post comparison by Wilcoxon signed-rank test. Significance: *P < 0.05.

### 3.4 Safety evaluation

During the entire trial period, no drug-related adverse reactions occurred in either the experimental group or the control group, and no serious adverse events happened in groups during the treatment process.

## 4 Discussion

As the aging process of China’s population accelerates, the incidence of CVD, especially angina pectoris events, has been increasing year by year, and the corresponding disease burden has been growing heavier day by day (Qu et al., 2024; Li et al., 2023). Currently, it has become a major health problem affecting the country, society, and individuals. At present, the primary goals of the treatment for angina pectoris are to improve myocardial blood supply and prognosis and to enhance the quality of life by reducing the symptoms of angina pectoris. These goals can be achieved through improving lifestyle, using preventive medications, and selectively applying coronary revascularization (Montone et al., 2024). The preventive medications commonly used in clinical practice to reduce the incidence of angina pectoris mainly include beta-blockers, calcium channel blockers, lipid-lowering drugs, antiplatelet drugs, nitrates, and so on (Zhang et al., 2023; Grundy et al., 2019). Although there is a wide variety of existing medications for the treatment of angina pectoris, it is still nitrates that can quickly relieve the immediate symptoms of angina pectoris. The choice of medications is rather limited (Virani et al., 2023). Therefore, it is of positive significance to seek more effective ways to relieve symptoms and increase the available treatment options.

This study provides preliminary yet important clinical evidence for the use of YXP in combination with conventional Western medicine in patients with SAP. In this randomized, non-inferiority, active-controlled trial, the intervention achieved a modest but statistically significant improvement—measured primarily by the SAQ—compared with standard therapy alone. The combination treatment was well tolerated, with no serious adverse events observed during the study period. Our results align with the growing body of evidence indicating the unique advantages of TCM in the treatment of angina pectoris. By acting on multiple targets and through numerous pathways, TCM interventions may alleviate angina symptoms, improve exercise tolerance, and enhance quality of life (Tian et al., 2024). Although our study has been limited by methodological weaknesses, the available literature suggests that similar Chinese botanical drug formulas can help relieve angina symptoms, improve quality of life, and enhance prognosis, indicating that integrative approaches combining Western and traditional medicine may confer additional benefits over conventional treatment alone.

From the perspectives of clinical service and evidence-based practice, our study highlights the importance of scientifically evaluating traditional medicine based on modern clinical research standards. Incorporating proven-effective traditional therapies into modern healthcare systems could expand therapeutic options for SAP, particularly for patients who are intolerant of or have limited access to Western pharmaceuticals. However, because this was a small-scale, non-inferiority trial, the reliability of the findings may be limited, as slight biases or random variations could easily influence the results. Therefore, our study should be regarded as a pilot trial. Further randomized controlled studies with larger samples, multicenter design, double-blinding, longer follow-up, and major cardiovascular and quality-of-life endpoints are essential to confirm our findings and clarify their generalizability.

From a mechanistic perspective, the pharmacological effects of YXP are attributed to a spectrum of bioactive metabolites derived from its constituent botanical drugs, such as ginsenosides (from Panax ginseng), bufadienolides (from Bufonis Venenum), eugenol (from Syzygium aromaticum), and bile acids (from Suis Fellis Pulvis). These metabolites exhibit complex synergistic pharmacological actions, including vasodilation, improvement of endothelial function, anti-inflammatory, and antioxidative properties (Kim, 2018; Wei et al., 2023; Lee et al., 2002). Compared with SBP, the main difference in the composition of YXP lies in the fact that it contains pig bile powder. Current research suggests that the main active metabolites in pig bile (such as bile acids, taurine, etc.) have been demonstrated to possess multiple pharmacological effects, including anti-inflammatory, lipid metabolism regulation, vascular endothelial protection, and antiplatelet aggregation (Li et al., 2022; Lefebvre et al., 2009). These modern pharmacological actions are consistent with the implications of improving microcirculation and reducing inflammatory damage in the traditional Chinese medicine theory of promoting blood circulation and removing toxins. Some of these effects have been demonstrated in basic and limited clinical studies. It is noteworthy that a growing body of evidence supports inflammation as a fundamental driver of residual risk in atherosclerotic cardiovascular disease (ASCVD), with a causal relationship to its development and progression (Hansson, 2005; Libby, , 2002). In this study, despite receiving standardized conventional Western medical treatment, the median hs-CRP level of participants at enrollment remained above the clinically recognized threshold of 1.0 mg/L, which indicates persistent residual inflammatory risk. After 1 month of combined treatment with YXP or SBP, the median hs-CRP levels in both groups decreased to below 1.0 mg/L. This change suggests that the addition of these Chinese patent medicines to standard therapy may further suppress systemic inflammation. These findings not only provide clinical evidence for the anti-inflammatory effects of both drugs in patients with stable angina pectoris but also hint at their potential value in managing residual cardiovascular inflammatory risk and improving long-term prognosis (Yu et al., 2024). As this study primarily focused on the clinical efficacy of YXP, its precise pharmacodynamic mechanisms, as well as its potential interactions with standard antianginal medications, remain to be elucidated. Future investigations employing network pharmacology, omics technologies, and mechanistic studies are warranted.

The limitations of this study should be noted. First, this study is a single-center exploratory trial with a relatively small sample size, and all patients were recruited from Dongzhimen Hospital, Beijing University of Chinese Medicine. This may limit the generalizability of the findings and introduce potential selection bias. Furthermore, as an exploratory study, the observed results may be subject to type I error risk due to multiple comparisons. Any efficacy signals identified in these analyses require validation in future large-scale confirmatory trials. Therefore, larger-scale, multi-center clinical trials are warranted to provide more robust and conclusive evidence. Secondly, due to the significant differences in color, shape, and odor between the two intervention drugs, a double-blind design could not be implemented in this study. Consequently, only a single-blind design (with blinding of participants only) was adopted. This methodological limitation may introduce performance or assessment bias. Future research should explore appropriate improvement strategies, such as employing a double-dummy technique or selecting objective hard endpoints, to mitigate this limitation. Finally, the long-term prognostic impact of the intervention requires further in-depth investigation. Future studies should aim to adopt double-blind methodologies where possible and explore the sustained effects of the treatment over extended follow-up periods.

All in all, YXP can effectively improve the clinical symptoms of patients with stable angina pectoris, reduce the blood lipid level and inflammation level of patients with angina pectoris, expand the range of medication options for clinical patients with angina pectoris, and provide new feasibility for the clinical treatment of such patients.

## Data Availability

The raw data supporting the conclusions of this article will be made available by the authors, without undue reservation.
