# Supplementary material for: Efficacy and safety of Yingxin pill for stable angina pectoris with heart blood stasis obstruction syndrome: a randomized, single-blind, positive-controlled trial
Source: Front Pharmacol. 2026 Feb 17;17:1730842. doi: 10.3389/fphar.2026.1730842 (PMC12953367; doi:10.3389/fphar.2026.1730842)
Supplement: Supplementary file 1 [file DataSheet1.pdf]

Supplementary Materials

1. Packaging of the investigational medicinal product.

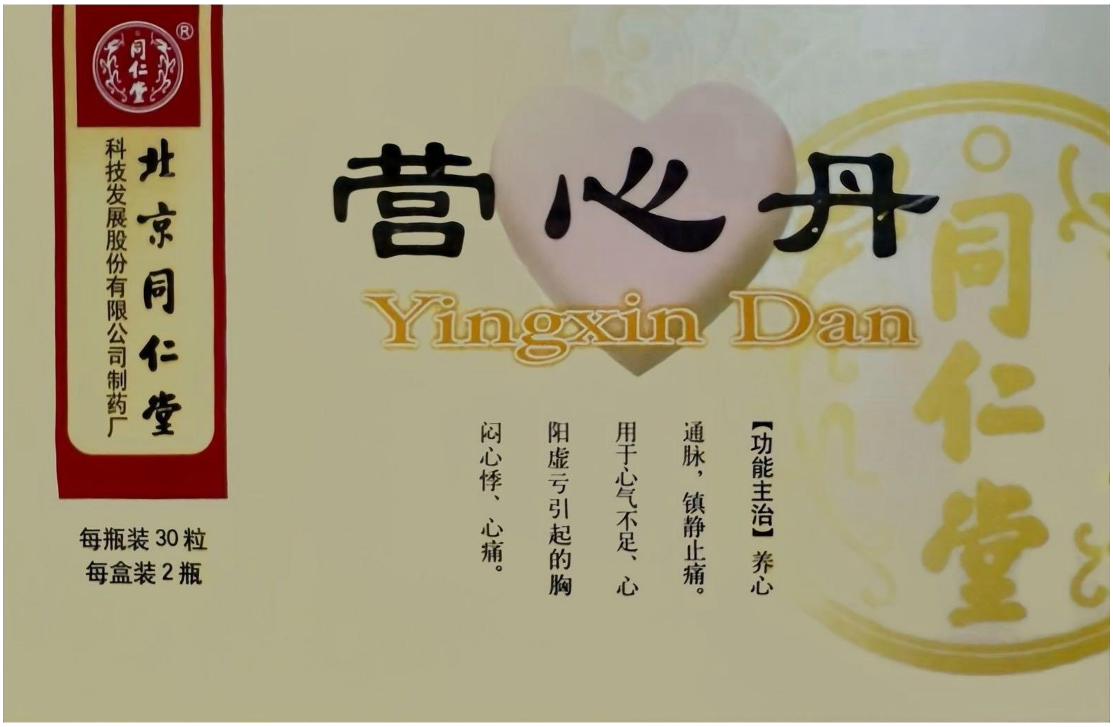

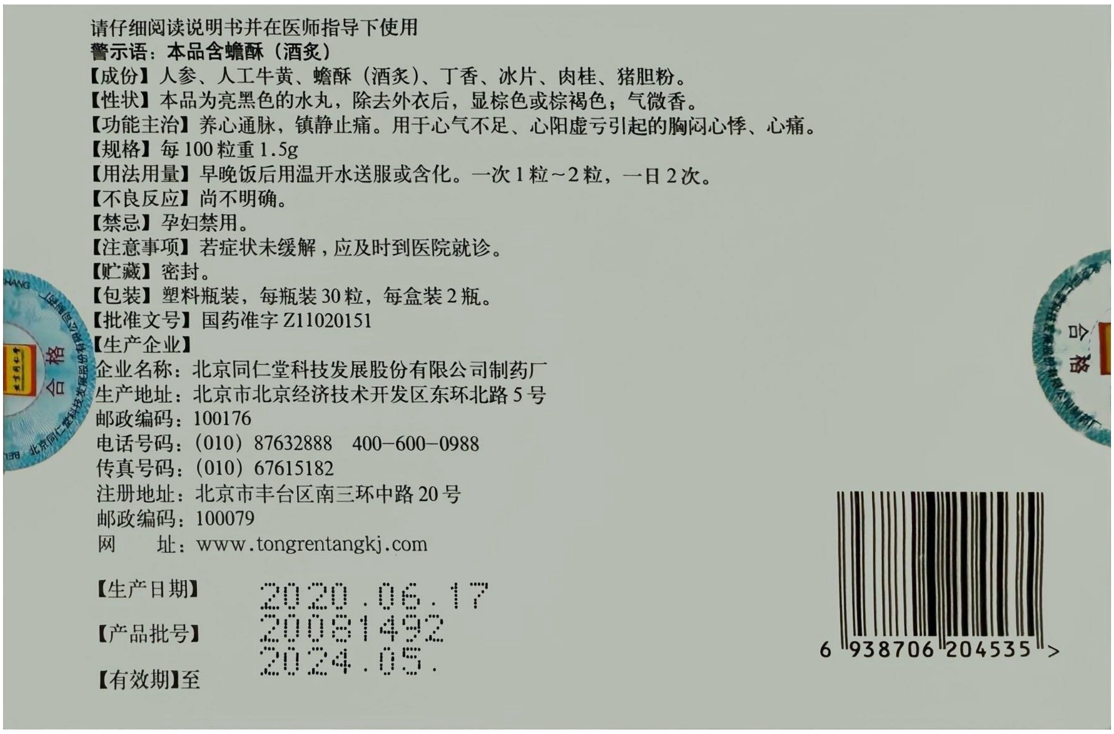

2. Package Insert of the Investigational Medicinal Product.

核准日期：2007年03月27日

修改日期：2013年12月30日

营心丹说明书

请仔细阅读说明书并在医师指导下使用

警示语：本品含蟾酥（酒炙）

【药品名称】

通用名称：营心丹

汉语拼音：Yingxin Dan

【成份】人参、人工牛黄、蟾酥（酒炙）、丁香、冰片、肉桂、猪胆粉。

【性状】本品为亮黑色的水丸，除去外衣后，显棕色或棕褐色；气微香。

【功能主治】养心通脉，镇静止痛。用于心气不足、心阳虚亏引起的胸闷心悸、心痛。

【规格】每100粒重1.5g

【用法用量】早晚饭后用温开水送服或含化。一次1粒～2粒，一日2次。

【不良反应】尚不明确。

【禁忌】孕妇禁用。

【注意事项】若症状未缓解，应及时到医院就诊。

【贮藏】密封。

【包装】塑料瓶装，每瓶装30粒。

【有效期】48个月

【执行标准】中华人民共和国卫生部药品标准中药成方制剂第十五册 WS_3-B-2989-98

【批准文号】国药准字Z11020151

【生产企业】

企业名称：北京同仁堂科技发展股份有限公司制药厂

生产地址：北京市北京经济技术开发区东环北路5号

邮政编码：100176

电话号码：（010）87632888　400-600-0988

传真号码：（010）67615182

注册地址：北京市丰台区南三环中路20号

邮政编码：100079

网　　址：www.tongrentangkj.com

3. The manufacturing standard for the investigational drug—Volume 15 of the Drug Standards for Proprietary Chinese Medicines, Ministry of Health of the People's Republic of China.

WS_3—B—2989—98

营 心 丹

Yingxin Dan

【处方】 人参　　人工牛黄　　蟾酥(酒炙)
丁香　　冰片　　肉桂
猪胆粉

【制法】 以上七味,除人工牛黄、冰片、猪胆粉外,蟾酥粉碎成细粉(单培在母子上),其余人参等三味加淀粉 60g 混匀,粉碎成细粉(取出适量起母子用),将冰片研细,与上述粉末配研,过筛,混匀,用水泛丸,低温干燥,用香墨-赭石(3∶1)粉末 10g 包衣,打光,即得。

【性状】 本品为亮黑色的水丸,除去外衣后,显棕色或棕褐色;气微香。

【鉴别】 (1)取本品,置显微镜下观察:草酸钙簇晶直径 20～68μm,棱角锐尖。石细胞类方形或类圆形,壁一边非薄。花粉粒三角形,直径约 16μm。

(2)取本品 1g,研细,加氯仿 20ml,超声处理 20 分钟,滤过,滤液蒸干,残渣加乙醇 0.5ml 使溶解,作为供试品溶液。另取脂蟾毒配基对照品,加氯仿制成每 1ml 含 1mg 的溶液,作为对照品溶液。照薄层色谱法(附录Ⅵ B)试验,吸取上述两种溶液 10μl,分别点于同一硅胶 G 薄层板上,以环己烷-氯仿-丙酮(4∶3∶3)为展开剂,展开,取出,晾干,喷以 5%香草醛硫酸溶液,热风吹至斑点显色清晰。供试品色谱中,在与对照品色谱相应的位置上,显相同的蓝绿色斑点。

(3)取胆酸对照品、猪去氧胆酸对照品,加乙醇制成每 1ml 各含 2mg 的混合溶液,作为对照品溶液。照薄层色谱法(附录Ⅵ B)试验,吸取[鉴别](2)项的供试品溶液与上述对照品溶液各 2μl,分别点于同一硅胶 G 薄层板上,以氯仿-乙醚-冰醋酸(2∶2∶1)为展开剂,展开,取出,晾干,喷以 10%硫酸乙醇溶液,在 105℃烘约 5 分钟,置紫外光灯(365nm)下检视。供试品色谱中,在与对照品色谱相应的位置上,显相同颜色的两个荧光斑点。

【检查】 应符合丸剂项下有关的各项规定(附录Ⅰ A)。

【浸出物】 照醇溶性浸出物测定法项下的热浸法(附录Ⅹ A)测定,用乙醇作溶剂,不得少于 15.0%。

【功能与主治】 养心通脉,镇静止痛。用于心气不足、心阳虚亏引起的胸闷心悸、心痛。

【用法与用量】 早晚饭后用温开水送服或含化,一次 1～2 粒,一日 2 次。

【规格】 每 100 粒重 1.5g

【贮藏】 密封。

注:猪胆粉的质量标准

本品为猪胆汁制成,以干燥品计算,含胆酸不得少于 45%。

[性状] 本品为棕黄色的粉末;气微腥,味苦。

[鉴别] 取本品约 5mg,加 60%醋酸 2ml 溶解后,加新制的糠醛溶液(1→100ml)2ml 与硫酸溶液(取硫酸 50ml,加水 65ml,混匀)13ml,在 70℃水浴中加热,即显蓝紫色。

[检查] 干燥失重 在 105℃干燥至恒重,减失重量不得过 10%(附录Ⅸ G)。

［浸出物］ 照醇溶性浸出物测定法项下的热浸法(附录Ⅹ A)测定，用乙醇作溶剂，按干燥品计算不得少于75%。

［含量测定］ 取本品约0.5g，精密称定，置锥形瓶中，加无水乙醇20ml，加热回流半小时，滤过，滤渣用适量无水乙醇洗涤，洗液与滤液合并，置水浴上蒸干，放冷，加乙醚50ml，搅匀，稍放置，滤过，弃去滤液，将滤纸连同滤渣放入原锥形瓶中，加15%氢氧化钠溶液和乙醇1ml，煮沸4小时，再加水30ml，滤过，滤液移至分液漏斗，容器用少量热水洗涤，洗液并入分液漏斗中，加硫酸使成酸性，放冷，用乙醚振摇提取4次，每次5ml，合并乙醚液，用水洗2次，每次10ml，洗液用少量乙醚振摇提取，合并乙醚液，置已干燥至恒重的蒸发皿中，于低温挥去乙醚，在105℃干燥至恒重，计算，即得。

本品以干燥品计算，含胆酸不得少于45%。

4. Herbarium Voucher Information for Reference Specimens of Key Botanical Drugs in Yingxin Pill (YXP).

**Medicinal Name (Chinese)**	**Botanical Drug Name (Latin)**	**Voucher Specimen Number**	**Herbarium (Acronym)**	**Collector(s)**	**Locality**	**Collection Date**
Panax ginseng C. A. Mey.	Ginseng Radix et Rhizoma	HNWP37118	Northwest Institute of Plateau Biology, CAS (HNWP)	Lin Hua, Li Huaci	Qinghai Province, China	Aug 1973
Syzygium aromaticum (L.) Merr. & L.M.Perry	Caryophylli Flos	IBSC 0513955	South China Botanical Garden, CAS (IBSC)	Lin Yourun	Hebei Province, China	03 Jun 1975
Neolitsea cassia (L.) Kosterm.	Cinnamomi Cortex	IBSC 0046208	South China Botanical Garden, CAS (IBSC)	Luo Xianrui	Hainan Province, China	Aug 1972

Note: The voucher specimens listed above serve as authoritative references confirming the taxonomic identity of the plant species corresponding to the botanical drugs used in the formulation of YXP. The materials actually administered in the present clinical trial were part of a single commercial batch (Lot No. 20081492) manufactured by Beijing Tongrentang Science and Technology Development Co., Ltd., in compliance with current pharmacopeial standards.
